# Microcephaly is associated with impaired educational development in children with congenital heart disease

**DOI:** 10.3389/fcvm.2022.917507

**Published:** 2022-10-06

**Authors:** Constanze Pfitzer, Laura K. Sievers, Alina Hütter, Hashim-Abdul Khaliq, Martin Poryo, Felix Berger, Ulrike M. M. Bauer, Paul C. Helm, Katharina R. L. Schmitt

**Affiliations:** ^1^Department of Congenital Heart Disease/Pediatric Cardiology, Deutsches Herzzentrum Berlin, Berlin, Germany; ^2^Berlin Institute of Health (BIH), Berlin, Germany; ^3^DZHK (German Centre for Cardiovascular Research), Partner Site Berlin, Berlin, Germany; ^4^Department of Internal Medicine I., Christian-Albrechts-University and University Hospital Schleswig-Holstein, Kiel, Germany; ^5^Department of Pediatric Cardiology, Saarland University Medical Center, Homburg, Germany; ^6^Department of Pediatric Cardiology, Charite – Universitaetsmedizin Berlin, Berlin, Germany; ^7^National Register for Congenital Heart Defects, Berlin, Germany; ^8^Competence Network for Congenital Heart Defects, Berlin, Germany

**Keywords:** microcephaly, congenital heart disease, education, school, supportive interventions, development

## Abstract

**Objectives:**

This study aims to evaluate the school careers of patients with congenital heart disease (CHD) and microcephaly.

**Methods:**

An exploratory online survey was conducted on patients from a previous study on somatic development in children with CHD in 2018 (*n* = 2818). A total of 750 patients participated in the online survey (26.6%). This publication focuses on 91 patients (12.1%) diagnosed with CHD and microcephaly who participated in the new online survey.

**Results:**

Microcephaly was significantly associated with CHD severity (*p* < 0.001). Microcephalic patients suffered from psychiatric comorbidity two times as often (67.0%) as non-microcephalic patients (29.8%). In particular, the percentage of patients with developmental delay, intellectual debility, social disability, learning disorder, or language disorder was significantly increased in microcephalic CHD patients (*p* < 0.001). A total of 85.7% of microcephalic patients and 47.6% of non-microcephalic patients received early interventions to foster their development. The school enrollment of both groups was similar at approximately six years of age. However, 89.9% of non-microcephalic but only 51.6% of microcephalic patients were enrolled in a regular elementary school. Regarding secondary school, only half as many microcephalic patients (14.3%) went to grammar school, while the proportion of pupils at special schools was eight times higher. Supportive interventions, e.g., for specific learning disabilities, were used by 52.7% of microcephalic patients and 21.6% of non-microcephalic patients.

**Conclusion:**

Patients with CHD and microcephaly are at high risk for impaired educational development. Early identification should alert clinicians to provide targeted interventions to optimize the developmental potential.

## Introduction

Children with congenital heart disease (CHD) are at risk of impaired somatic growth, especially those with single ventricle physiology (1, 2). The cause of growth retardation is complex and multifactorial, with genetic and hemodynamic factors as essential determinants for the underlying CHD (3, 4).

Most studies analyzing somatic development focus on body weight and length but less often on the head circumference (2, 4, 5). However, head circumference is an important somatic parameter, as microcephaly may indicate impaired neurodevelopmental outcomes from early childhood until adolescence (6–9). Neurodevelopmental deficits may have a broad clinical manifestation and may also affect areas of memory and executive function, visual-spatial imagination, attention, and social skills (10) and therefore impair the child's school career.

To date, the association between microcephaly and education in children with CHD has not been analyzed. Hence, this study aimed to evaluate the scholastic development of CHD patients with microcephaly.

## Patients and methods

### Study design and setting

This is an exploratory cross-sectional follow-up study using an online survey to evaluate the school careers of patients with CHD. The ethics committee of the Charité—Universitätsmedizin Berlin, Germany, approved this study (no. EA2/190/19), which was conducted in 2020.

Study participants received an invitation to participate in the survey by email or postal letters, and if a response was missing, they were reminded once to participate. The survey could be completed by the patient, a parent, or a third party (legal guardian or caregiver). The corresponding addressee received a slightly different questionnaire. The survey consisted of a maximum of 74 questions, depending on the participant and the option of sequential questions. A total of 92% of the survey's questions were closed multiple-choice questions. Most questions offered the participant the option of adding their own answer if none of the possible answers adequately described their situation in a free-text box. The survey comprised questions about medical data such as further chronic diseases, psychiatric comorbidities (attention deficit disorder, developmental disability, intellectual debility, social disability, emotional disability, depression, anxiety disorder, learning disorder, and language disorder), genetic syndromes, and subjective health conditions. The focus was on questions about the school career: enrollment age, school form (primary and secondary), (early) supportive therapy (physiotherapy, speech therapy, occupational therapy, or psychotherapy), school year repetition, absenteeism from school, and participation in physical education. Furthermore, the educational achievement and qualifications of the parents were elicited. Medical data were supplemented by the database of the National Register for Congenital Heart Defects (NRCHD). The NRCHD is Germany's national repository for medical data on CHD patients. With ~55,000 members, the NRCHD is Europe's largest register for CHD patients and can be regarded as a basis for representative studies (11).

### Patient cohort

From 2006 to 2011, the Competence Network for Congenital Heart Defects (Berlin, Germany) conducted the “PAN-study” (Prävalenz angeborener Herzfehler bei Neugeborenen in Deutschland), which analyzed the prevalence of CHD prospectively in newborns in Germany (12). To determine the somatic development (height, length, and head circumference) of the PAN-cohort, the “PANKU-study” (Prävalenz angeborener Herzfehler bei Neugeborenen in Deutschland Kopfumfang) was carried out; a follow-up study analyzed somatic developmental data from the “Kinderuntersuchungsheft” (“child's medical records”), which records the examination results of the mandatory screening for children and adolescents (13). To assess the academic development of this particular cohort, we chose this cohort for the present study “PANKU-Education” (Prävalenz angeborener Herzfehler bei Neugeborenen in Deutschland: Kopfumfang—Education). Of the 2818 families that were contacted, 750 (26.6%) completed the survey.

#### Inclusion criteria

- Participation in the “PAN-study” and “PAN-KU-study”
- Availability of complete and up-to-date contact information at the time of the present study
- Complete answering the online-survey

### Microcephaly

To analyze the prevalence of microcephaly in the presented study, head circumference data at the child's three-month checkup (corresponding to U4 screening between the 3rd and 4th month of life in the German health care system) were used. These data were converted into sex- and age-adjusted percentiles, taking into account the date of measurement, date of birth, sex, and gestational age at birth. The percentiles, according to Braegger et al., commonly used in Germany, served as the basis for the calculation (14). Microcephaly was defined as a head circumference < 3^rd^ percentile.

### The german school system

In Germany, school attendance is compulsory until the age of 15 years and is free. Usually, children start elementary school at the age of 5–7 years, mainly, however, at the age of 6 years. If a child has special needs in their educational, developmental, and learning possibilities (e.g., due to a learning or mental/cognitive disability, a sensory and/or physical disability, or less frequently due to a long-term illness), they are introduced to a special school. After finishing primary education (4–6 years, depending on the federal state), there are several options for secondary schooling according to the student's abilities: the highest is the grammar school (“Gymnasium”), where pupils graduate after 8–9 years with a high school diploma, enabling them to study at university. Graduation from secondary school options (usually after 5–7 years) allows for starting an apprenticeship.

### Statistical analyses

The cardiac diagnoses were arranged in accordance with the classification of the International Pediatric and Congenital Cardiac Code (IPCCC) (15). Following Warnes et al., the CHD diagnoses were assigned to four groups: simple CHD, moderate CHD, complex CHD, and other/non-classified CHD (16).

For the various research questions, different subgroups were defined. The influence of parental education level was analyzed using descriptive analysis due to limited data.

For this, the parental education level was classified as “high,” “medium,” and “low” according to the International Standard Classification of Education (ISCED) (17). Group differences were analyzed using the chi-square test for nominal variables of independent subsamples. The Mann-Whitney U test was used for ordinal scaled variables, and interval scaled variables were analyzed using the *t*-test. A *p*-value of < 0.05 was considered statistically significant. SPSS (Statistics for Mac, Version 27.0, IBM Crop. Armonk, NY) was used for statistical analysis.

## Results

### Description of the overall cohort

A total of 750/2818 study participants (26.6%) answered all survey questions and were included in the statistical analyses. The mean age of the overall study cohort was 12.16 years (confidence interval: 12.10–12.22), and 50.4% were females (Table 1). Subjective health status was excellent, but psychiatric comorbidity was reported in 34.5%. Most patients had simple CHD (51.3%), with ventricular septal defect (40.7%) and atrial septal defect (11.9%) being the most frequent ones (Table 2).

**Table 1:** Characteristics of all patients from our cohort.

	***N***		**Mean**	**SD**
			**Median**	**IQR**
Total participants	750	100%		
Mean age			12.16	0.847
Sex	
Female	378	50.4%		
Male	372	49.6%		
Health status	
Subjective health status (1 very good- 6 bad)			*1*	*1*
Epilepsy	13	1.7%		
Optic support	262	34.9%		
Hearing support	22	2.9%		
Walking aid	11	1.5%		
Psychiatric comorbidity	259	34.5%		
Gestational age	
Preterm	115	15.8%		
Term	610	83.8%		
Post-term	3	0.4%		
**Syndromic disorders**	64	8.3%		
Alagille syndrome	1	1.6%		
DiGeorge syndrome	5	7.8%		
Goldenhar syndrome	2	3.1%		
Trisomy 18	1	1.6%		
Trisomy 21	38	59.4%		
Noonan syndrome	3	4.7%		
Williams syndrome	3	4.7%		
Others	11	17.2%		

**Table 2:** CHD diagnoses of the patients in our cohort.

	***N***	
Ventricular septal defect	305	40.7%
Atrial septal defect	89	11.9%
Tetralogy of Fallot	48	6.4%
Univentricular heart	46	6.1%
Aortic valve disease	36	4.8%
Coarctation of the aorta	35	4.7%
Transposition of the great arteries (intact ventricular septum)	32	4.3%
Atrioventricular septal defect	29	3.9%
Pulmonary valve stenosis	28	3.7%
Patent ductus arteriosus	20	2.7%
Other	82	10.9%

**Figure 1:**
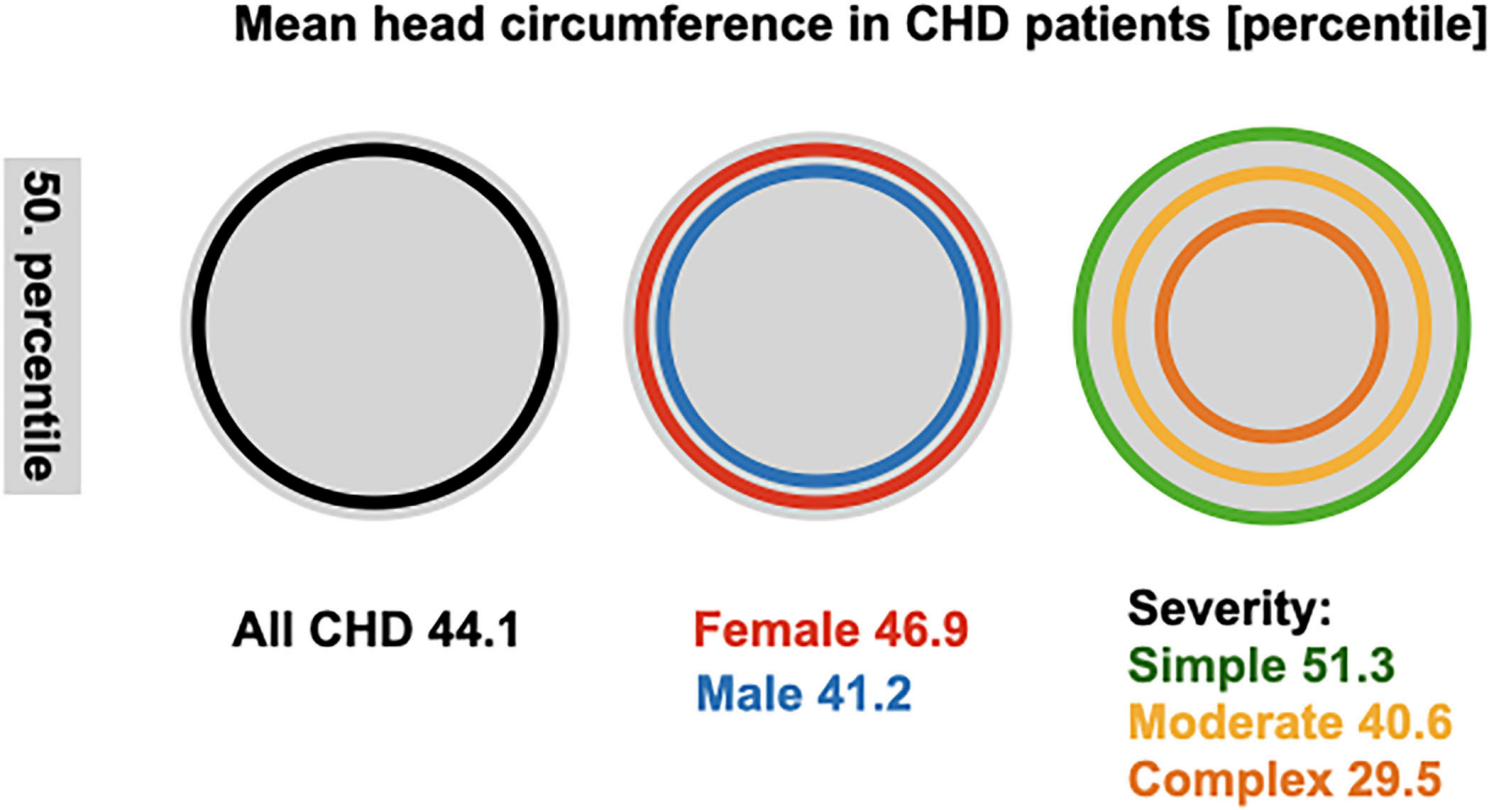
Mean head circumference in CHD patients. Head circumference percentiles at 3–4 months in all CHD patients and subgroups. The gray circle represents the 50 percentile, and data of patients and subgroups are denoted as the mean. SD for all, sex and simple severity are 33, moderate severity 32, and complex 31.

At the age of 3–4 months, the mean head circumference of CHD patients from our cohort (percentile 44.1 ± 33.3) equaled the age-dependent percentile; Figure 1 illustrates the head circumference percentiles. The mean percentile in female patients was 46.9 ± 33.3 and in male patients 41.2 ± 33.1. Patients with simple CHD had a head circumference approximating the 51.3 ± 33.8 percentile, moderate CHD 40.6 ± 33.1, and complex CHD 29.5 ± 33.2.

### Characterization of microcephalic CHD patients

In total, 91 out of 750 patients (12.1%) had a head circumference below the 3rd percentile and thus were classified as microcephalic (Tables 3, 4). This subgroup had a mean age of 12.2 ± 0.85 years; 48.4% were female, and 51.6% were male. Their subjective health was not as good as the non-microcephalic patients' but still very good (51.6% of microcephalic vs. 66.3% of non-microcephalic patients rated their health status as very good) (Table 3). The prevalence of epilepsy and the need for optic support, hearing support, or walking aid was higher among microcephalic patients. In contrast, gestational age was not significantly associated with microcephaly.

**Table 3:** Characteristics of microcephalic and non- microcephalic patients.

	**Non-microcephalic patients**				**Microcephalic patients**				***p***
	***N***		**Mean**	**SD**	***N***		**Mean**	**SD**	
			**Median**	**IQR**			**Median**	**IQR**	
Total participants	621	100%			91	100%			
Mean age			12.14	841			12.2	0.885	n.s.
Sex			
Female	317	51.0%			44	48.4%			n.s.
Male	304	49.0%			47	51.6%			n.s.
Health status			
Subjective health status (1 very good- 6 bad)			*1*	*1*			*1*	*1*	+
Epilepsy	6	1.0%			7	7.7%			*
Optic support	199	32.0%			44	48.4%			+
Hearing support	8	1.3%			11	12.1%			*
Walking aid	4	0.6%			7	7.7%			*
Psychiatric comorbidity	185	29.8%			61	67.0%			*
Gestational age			
Preterm	94	15.1%			16	17.6%			n.s.
Term	524	84.4%			75	82.4%			n.s.
Post-term	3	0.5%			0	0%			n.s.
**Syndromic disorders**	32	5.2%			28	30.8%			*
Alagille syndrome	1	3.1%			-	-			
DiGeorge syndrome	2	6.3%			2	7.1%			
Goldenhar syndrome	1	3.1%			1	3.6%			
Trisomy 18	–	–			1	3.6%			
Trisomy 21	18	56.3%			17	60.7%			
Noonan syndrome	–	–			1	3.6%			
Williams syndrome	2	6.3%			1	3.6%			
Others	8	25.0%			5	17.9%			

*p* ^*^denotes significant difference compared to non-microcephalic *p* < 0.001, + denotes *p* < 0.05, *n.s*. = not significant.

For ordinal scaled variables, median and IQR were given accordingly (printed in italics).

**Table 4:** CHD diagnoses of microcephalic patients.

	***N***	
Ventricular septal defect	29	31.9%
Atrial septal defect	7	7.7%
Tetralogy of Fallot	6	6.6%
Univentricular heart	11	12.1%
Aortic valve disease	2	2.2%
Coarctation of the aorta	4	4.4%
Transposition of the great arteries (intact ventricular septum)	7	7.7%
Atrioventricular septal defect	12	13.2%
Other	13	14.3%

**Figure 2:**
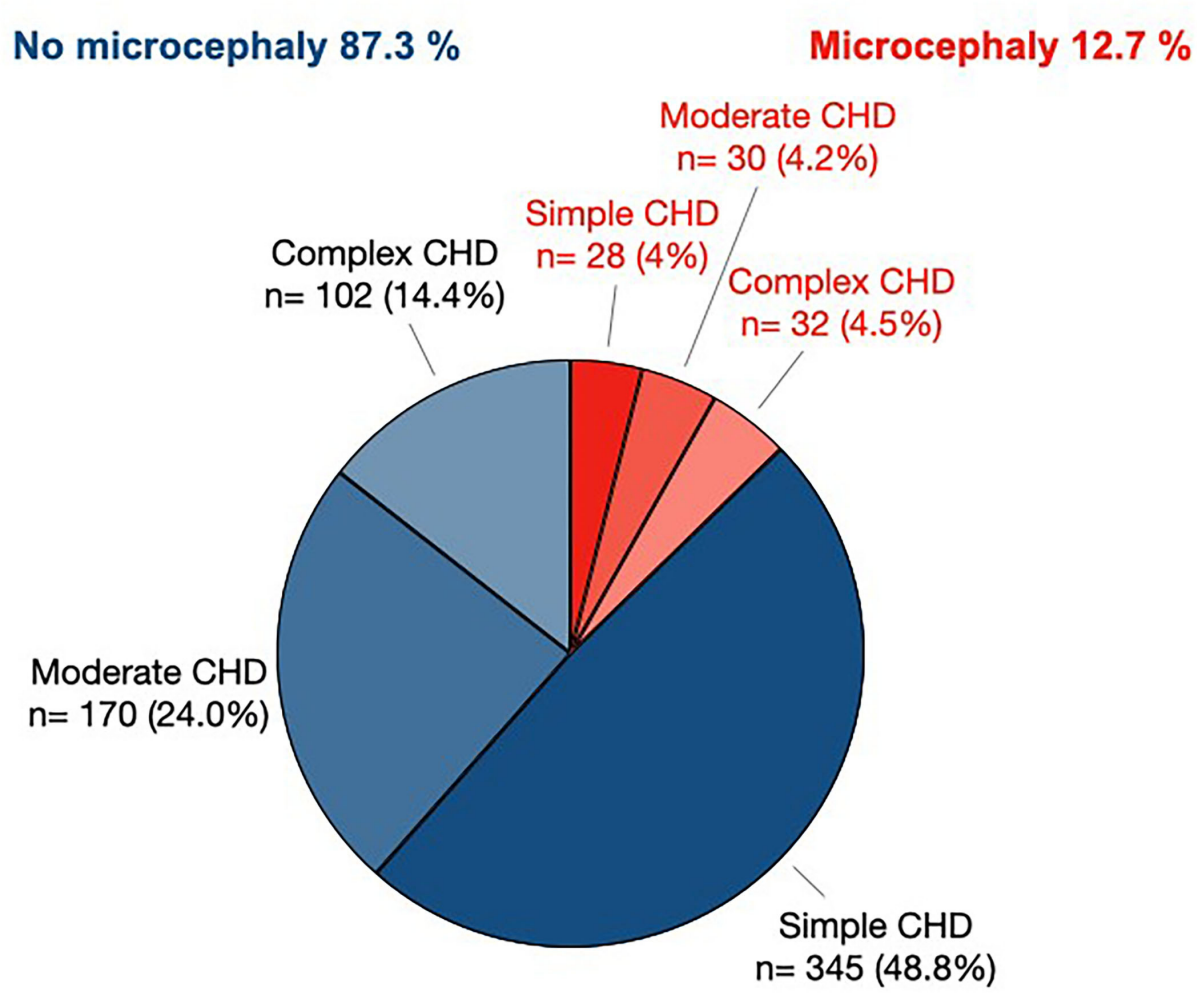
Prevalence of microcephaly in patients with simple, moderate, or complex CHD. The pie chart denotes the raw *n* of patients defined by CHD severity classification and head circumference. Only patients with known CHD severity scores and known head circumference are included (*n* = 707).

Syndromal diseases existed in 30.8% of the microcephalic patients (Table 3). Ventricular septal defect (31.9%), atrioventricular septal defect (13.2%), and univentricular heart (12.1%) were the most frequent CHDs in this subset (Table 4). Microcephaly was significantly associated with CHD severity (Figure 2). Simple CHD was seen in 55.9% of normocephalic patients, while moderate CHD was seen in 27.5% and complex CHD in 16.6%. In contrast, simple, moderate, and complex CHD was found in equal parts (31.1, 33.3, and 35.6%) in microcephalic patients.

**Figure 3:**
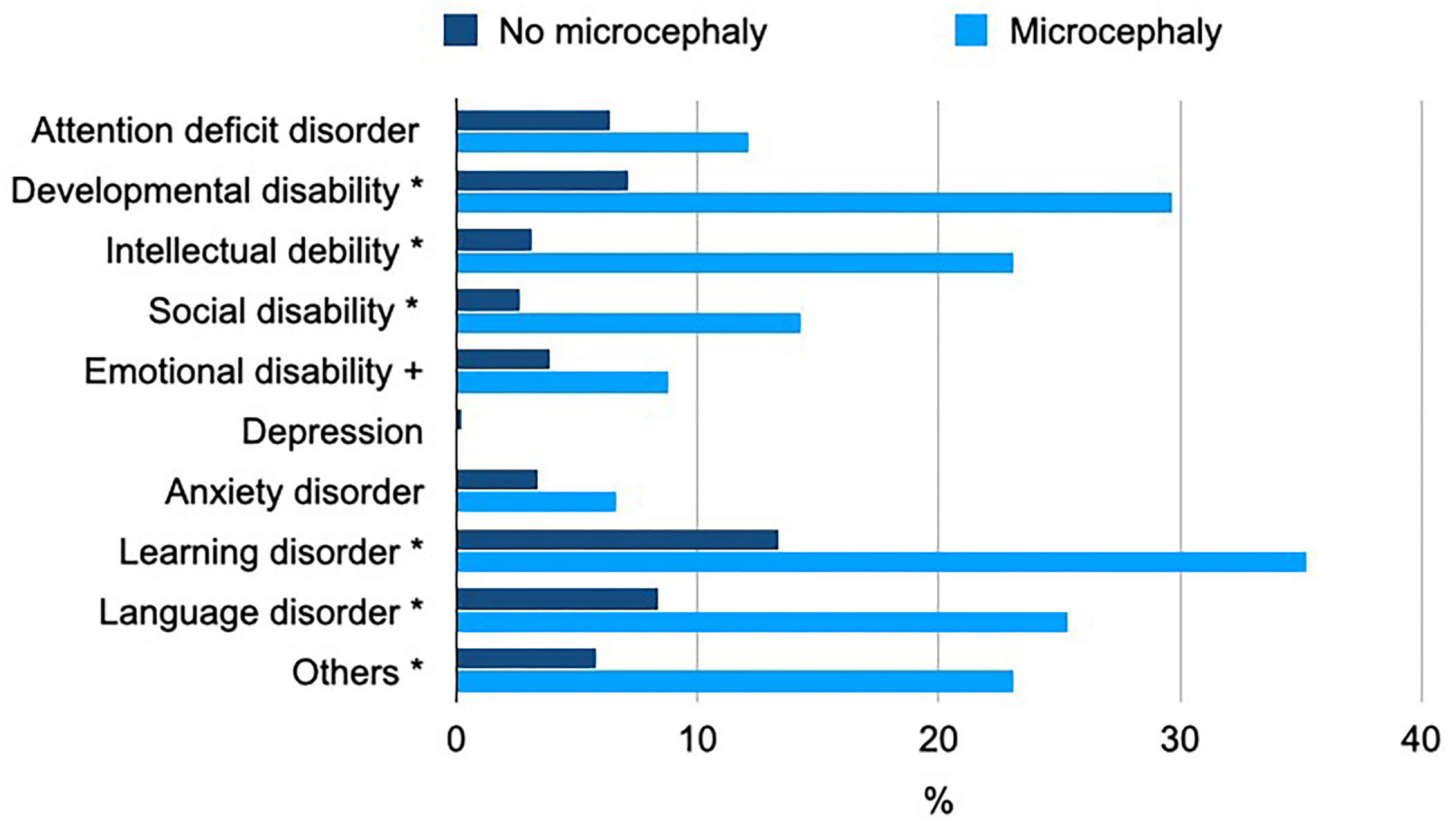
Psychiatric comorbidity in microcephalic CHD patients. The chart visualizes the percentage of patients with the most prevalent psychiatric disorders among microcephalic or non-microcephalic patients; * denotes a significant difference compared to non-microcephalic, *p* < 0.001, and + denotes *p* < 0.05.

A total of 29.8% of non-microcephalic patients suffered from psychiatric comorbidity, while in microcephalic patients, this proportion was more than two times as high (67.0%) (Table 3). In particular, the percentage of patients with developmental delay, intellectual debility, social disability, learning disorder, or language disorder was significantly increased in microcephalic CHD patients (*p* < 0.001; Figure 3).

### Educational status of microcephalic CHD patients

**Figure 4:**
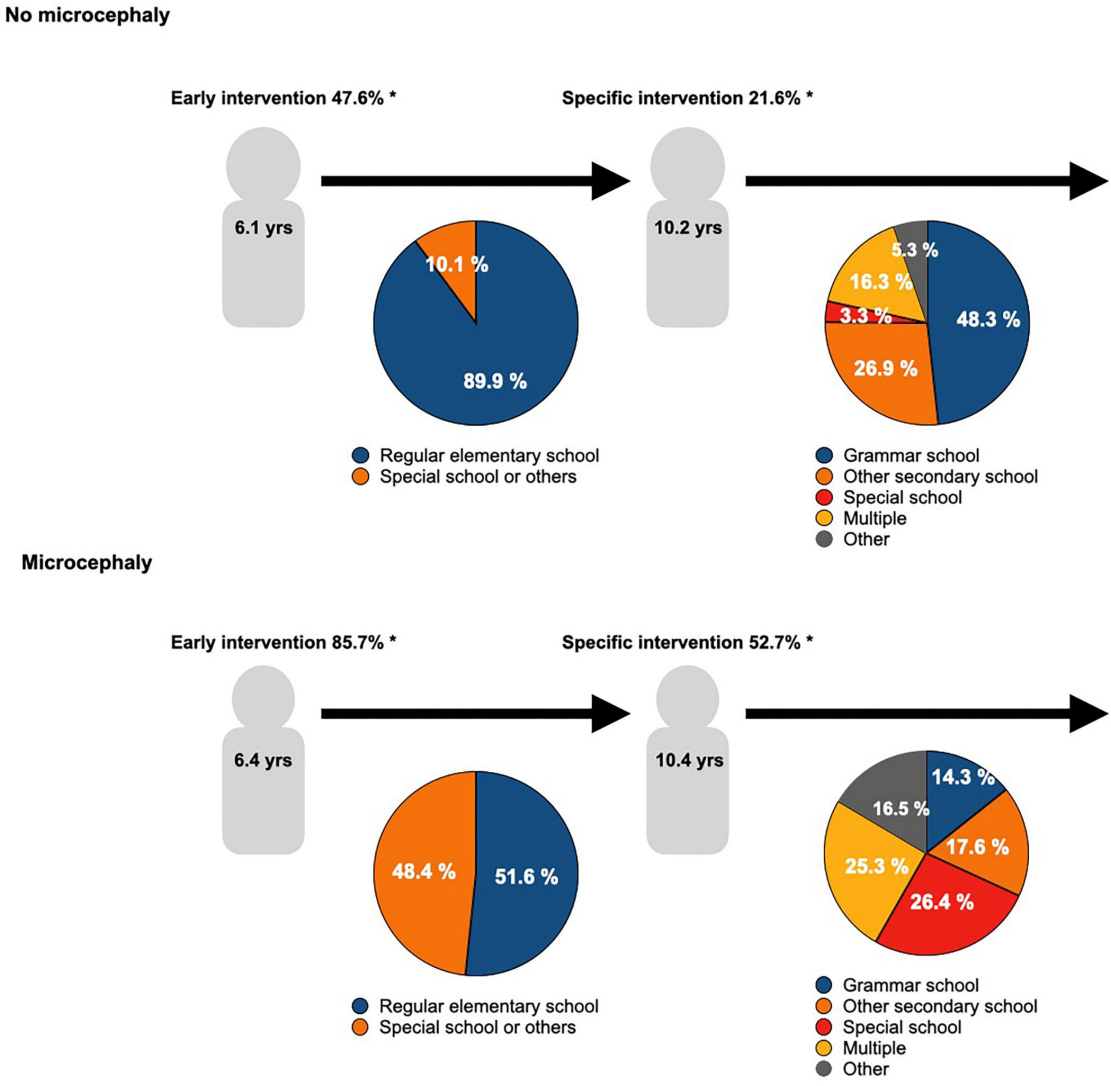
Educational development in CHD patients. The graph visualizes the school career of CHD patients with or without microcephaly. The median age of school enrollment and the first change of school are given in the figures. The distribution of different kinds of primary schools in the left pie chart is represented, and the pie chart on the right represents the different kinds of secondary schools. Further, the usage of early and/ or specific interventions to support the school careers is given. Differences in the kind of school are significant < 0.001; additionally * denotes a significant difference compared to non-microcephalic patients, *p* < 0.001, and + denotes *p* < 0.05.

The educational career and usage of support interventions of microcephalic patients compared to CHD patients without microcephaly are visualized in Figure 4. A total of 85.7% of the microcephalic patients but only 47.6% of non-microcephalic patients received early interventions to foster their development. The school enrollment of both groups was similar at approximately six years of age. 89.9% of non-microcephalic but only 51.6% of microcephalic patients were enrolled in a regular elementary school. About four years later, at the age of 10, children from both groups transferred to a new school: 48.3% of the non-microcephalic patients went to grammar school, and 3.3% needed specialized schools. In contrast, only 14.3% of the microcephalic patients went to grammar school, while the proportion of pupils at special schools was eight times higher than in non-microcephalic patients. Supportive interventions, e.g., because of specific learning disabilities, were used by 52.7% of microcephalic patients and 21.6% of non-microcephalic patients.

Staying down a year was statistically insignificant between microcephalic and non-microcephalic patients (Table 5); school grades were similar in both groups, too. Absenteeism from school added to < 1 month in most microcephalic patients (Table 5). In our cohort, parental educational level did not influence the attended school forms (primary and secondary school), the class repetition rate, or the utilization of (early) supportive measures.

**Table 5:** School career of the microcephalic patients.

	**Microcephaly (*****n*** = **91)**		**No microcephaly (*****n*** = **621)**		
	***N***		***N***		***p***
**Stayed down?**					
Yes	14	15.4 %	57	9.2 %	n.s.
No	77	84.6 %	564	90.8 %	
**Participating in physical education?**					
Yes	69	75.8 %	550	88.6 %	+
No	22	24.2 %	71	11.4 %	
**Absenteeism from school**					
< 1 month	73	82 %	552	94 %	*
1 month−1 year	16	18 %	33	5.6 %	
>1 year	0	0 %	2	0.3 %	

The table informs about school attendance in microcephalic and non-microcephalic patients. The whole cohort comprises 750, but data on the head circumference are missing in 28 patients. Thus, the sum of microcephalic and non- microcephalic patients is 712. ^*^ denotes significant difference compared to non-microcephalic *p* < 0.001, + denotes *p* < 0.05, n.s. = insignificant data regarding missed school time was missing in *n* = 2.

## Discussion

Our data demonstrate that microcephaly in early childhood is a crucial risk factor for impaired scholastic development in patients with CHD. These results expand the current state of research, as the educational career of microcephalic vs. non-microcephalic CHD patients has not been evaluated. This is noteworthy as education is a particularly important and objectively measurable characteristic of neurodevelopment. In our study, microcephalic participants had a high demand for early and specific interventions. They frequently attended special schools more frequently and compared to non-microcephalic CHD patients, their likelihood of attending a grammar school was halved. Approximately two-thirds of microcephalic patients were diagnosed with psychiatric disorders such as learning, emotional, or behavioral disorders.

Previous studies have shown that a small head circumference in CHD patients is related to neurodevelopmental outcomes in early childhood (6, 7, 18), preschool age (19), and adolescence (20). This is in line with our study results, as microcephalic CHD patients were confronted with multiple problems in their whole (pre-) scholastic development. The etiology of this phenomenon is complex and thought to be multifactorial.

First, mounting evidence suggests that prenatal factors play a crucial role. It has been proven that intrauterine brain growth is predominantly altered in children with complex CHD and may result in a smaller head circumference and brain volume at birth (21, 22). Hemodynamic factors are assumed as an explanatory variable and include umbilical vein oxygen saturation, altered blood flow, and reduced oxygen content in the ascending aorta (23). Moreover, animal and human studies suggest that prenatal maternal psychosocial stress may adversely affect the developing fetal brain (24, 25). However, for expectant mothers after the diagnosis of CHD in their child, this association has hardly been studied (26). A longitudinal study analyzing this would be even more important as a prenatal CHD diagnosis may be a traumatic situation for the expectant mother.

Second, postnatal growth is often impaired in CHD patients. This has mainly been attributed to disrupted normal feeding behavior in the neonatal and infant period (27). In CHD patients requiring early operative treatment, this problem often persists during the postoperative phase, as patients frequently require a nasogastric feeding tube (28, 29). Hypermetabolic state and malabsorption may be further pathophysiologic mechanisms, while genetic and environmental factors are thought to play a subordinate role (27, 30). These are important considerations when interpreting our study results because more than two-thirds of microcephalic CHD patients suffer from moderate or complex CHD.

Third, social environment in the meaning of parental education and supportive therapy may be a rationale as this is an important determinant in childhood development (31, 32). In our study cohort, almost all microcephalic patients received early supportive therapy, and specific interventions fostered more than half at school age. Supportive therapy may compensate for learning deficits (33) and contribute to the fact that a small proportion of microcephalic patients were able to attend grammar school. Interestingly, the parental educational level did not influence the child's scholastic development in the present analysis. This argues for the assumption that in this specific patient cohort, the neurodevelopmental outcome is predominantly associated with somatic factors. Preterm birth, a known risk factor for altered brain growth (34), did not significantly contribute to microcephaly in our study cohort as more than 80% of study participants were born at full term.

Another noteworthy result of the present analysis is the prevalence of psychiatric comorbidities: nearly one-third of non-microcephalic patients suffered from psychiatric comorbidity, but in microcephalic patients, this proportion was more than two times as high. Numerous studies have observed the clinical manifestation of developmental delay, intellectual debility, social disability, learning disorder, or language disorder (35, 36). Underlying pathophysiologic mechanisms are still under investigation. However, prenatal hemodynamic factors and a genetic link between heart and brain development are assumed to be important rationales (37).

Interestingly, the subjective health status of microcephalic patients was not as good as the non-microcephalic patients' but still very good. This has been reported before (38). A possible explanation could be that, as CHD patients were born with heart disease, they might have learned early in life how to develop a strong “sense of coherence” and select the right coping strategies (39).

### Clinical implications for this study

It is important for CHD patients, affected families, and treating physicians to be aware of microcephaly as a risk factor for impaired scholastic development. Routine follow-up examinations should be established to identify developmental deficits. Unfortunately, underlying variables such as prenatal hemodynamic factors or the number and point of time of CHD operations are hardly modifiable. However, supportive therapy seems to be a promising compensation mechanism. Therefore, CHD patients and their families should be given low-threshold access to supportive interventions.

### Limitations

Due to the data privacy policy of the NRCHD, a non-responder analysis could not be performed. Our results need to be interpreted in light of a potential selection bias. Highly educated and/or healthier CHD patients might be more inclined to participate in scientific studies than patients with lower educational levels and/or more health problems. However, a proportion of 48% of microcephalic patients attending a particular school speaks against this assumption. Our analysis of parental ISCED and child education supports this assumption that in microcephalic patients, somatic (co-) morbidities have a more significant influence than the parental level of education. However, parents with higher education might be overrepresented compared to the German general population. The response rate of 26.6% is in the middle range and may still be considered valid and quite representative (40). The results cannot be easily applied to other countries, as education and health care systems may differ. Due to the study setting (follow-up study of a patient cohort on the somatic development in children with CHD), we could not assess the highest educational/academic achievement as the mean age of our study cohort was ~12 years of age. Seventy patients in the study were diagnosed with a syndromal disease; among these, 28 were microcephalic. Based on the heterogeneity of diagnoses and small subgroups, we could not correct our results for this or perform subgroup analyses. Our study does not answer whether microcephalic patients with or without CHD differ in their educational perspectives. Microcephaly appears to be a risk factor for impaired educational success in CHD patients.

## Conclusion

Patients with CHD and microcephaly are at risk for impaired educational development. Head circumference measurement in infants and children with CHD should be integrated into the serial routine monitoring of somatic parameters in children with CHD. Microcephaly in early childhood should alert clinicians to provide targeted interventions to optimize the developmental potential. Further studies are necessary to evaluate the impact of these interventions and to determine the long-term follow-up of this specific patient cohort at risk.

## Data availability statement

The raw data supporting the conclusions of this article will be made available by the authors, without undue reservation.

## Ethics statement

The studies involving human participants were reviewed and approved by Ethics Committee of the Charité—Universitätsmedizin Berlin, Germany (No. EA2/190/19). Written informed consent to participate in this study was provided by the participants' legal guardian/next of kin.

## Author contributions

CP, H-AK, MP, FB, UB, PH, and KS: study concept/design. CP, PH, and KS: data interpretation. CP: drafting of the original manuscript. LS and AH: data analysis. AH: acquisition of data. LS: interpretation, visualization, and drafting of the original manuscript (results). PH, H-AK, MP, and KS: critical revisions of the manuscript. H-AK and MP: acquisition of data. FB and UB: critical revisions of the manuscript. All authors approved the final version of the manuscript.

## Funding

This study received funding from Deutsche Herzstiftung e.V. The funder was not involved in the study design, collection, analysis, interpretation of data, the writing of this article or the decision to submit it for publication. CP is participant in the BIH Charité Clinician Scientist Program funded by the Charité—Universitätsmedizin Berlin and the Berlin Institute of Health. This work was supported by the Competence Network for Congenital Heart Defects (Federal Ministry of Education and Research/grant number 01GI0601) and the National Register for Congenital Heart Defects (Federal Ministry of Education and Research/grant number 01KX2140). We acknowledge financial support by Land Schleswig-Holstein within the funding program Open Access Publikationsfonds.

## Conflict of interest

Authors UB and PH were employed by the companies National Register for Congenital Heart Defects and Competence Network for Congenital Heart Defects.

The remaining authors declare that the research was conducted in the absence of any commercial or financial relationships that could be construed as a potential conflict of interest.

The reviewer JL declared a shared affiliation with the author LS to the handling editor at the time of review.

## Publisher's note

All claims expressed in this article are solely those of the authors and do not necessarily represent those of their affiliated organizations, or those of the publisher, the editors and the reviewers. Any product that may be evaluated in this article, or claim that may be made by its manufacturer, is not guaranteed or endorsed by the publisher.
