# Supplementary material for: Microcephaly is associated with impaired educational development in children with congenital heart disease
Source: Front Cardiovasc Med. 2022 Oct 6;9:917507. doi: 10.3389/fcvm.2022.917507 (PMC9584804; doi:10.3389/fcvm.2022.917507)
Supplement: Supplementary file 1 [file Table_1.docx]

**Supplementary Table 1: ISCED-level of parents of non- and microcephalic patients**

|  | **No microcephaly** | | **Microcephaly** | |
| --- | --- | --- | --- | --- |
|  | **N** |  | **N** |  |
| Maternal ISCED |  |  |  |  |
| Low | 17 | 2,8 % | 4 | 4,4 % |
| Medium | 272 | 45,2 % | 43 | 47,8 % |
| High | 313 | 52,0 % | 43 | 47,8 % |
| Paternal ISCED |  |  |  |  |
| Low | 11 | 1,8 % | 3 | 3,3 % |
| Medium | 227 | 37,7 % | 41 | 45,6 % |
| High | 365 | 60,6 % | 47 | 52,2 % |
