# Supplementary material for: Microcephaly is associated with impaired educational development in children with congenital heart disease
Source: Front Cardiovasc Med. 2022 Oct 6;9:917507. doi: 10.3389/fcvm.2022.917507 (PMC9584804; doi:10.3389/fcvm.2022.917507)
Supplement: Supplementary file 2 [file Data_Sheet_1.PDF]

Teilnehmer-Login

Um an der Umfrage teilnehmen zu können, gib bitte hier den neunstelligen Login-Code ein, den wir per E-Mail/Post zugeschickt haben:

Wer beantwortet unsere Umfrage?

- ◯ Patient (Ich habe einen angeborenen Herzfehler).
- ◯ Elternteil (Mein Kind hat einen angeborenen Herzfehler).
- ◯ weder Patient noch Elternteil, sondern (z. B. Oma, Bruder/Schwester, Betreuerin etc.):

Wichtiger Hinweis

Da sich diese Umfrage direkt auch an Kinder und Jugendliche mit angeborenem Herzfehler richtet, wird in den Fragen zur einfachen Lesbarkeit die Anrede „Du" verwendet.

*Wenn Sie angegeben haben, dass Sie selbst **keinen angeborenen Herzfehler** haben, beantworten Sie die Frage bitte aus Patientensicht. Sie helfen uns damit, unsere Umfrage auf einen Fragebogen für alle zu beschränken. Das erspart uns viel Aufwand. Vielen Dank! Es ist wirklich ganz einfach, keine Sorge.*

Hier ein Beispiel

*Der Vater von Anna füllt den Fragebogen alleine, also stellvertretend für Anna, aus. Dazu wählt er bei der Frage „**Welches Geschlecht hast Du?**" die Antwort „weiblich" aus. Wenn Anna den Fragebogen allein ohne ihren Vater beantwortet (weil Anna schon alt genug ist), wählt sie ebenfalls „weiblich" als Antwort aus.*

Welches Geschlecht hast Du?

Bitte Zutreffendes ankreuzen.

- ◯ Männlich.
- ◯ Weiblich.
- ◯ Divers.

Wie alt bist Du?

Bitte gib Dein Alter in Jahren an.

- ◯ Ich bin (Alter in Jahren):

Wie geht es Dir derzeit gesundheitlich?

Bitte beurteile Deinen derzeitigen Gesundheitszustand auf einer Skala von 1 (sehr gut) bis 6 (gar nicht gut).
Wird der Fragebogen stellvertretend ausgefüllt, beurteilen Sie bitte den derzeitigen Gesundheitszustand des Kindes.

1 (sehr gut)	2	3	4	5	6 (gar nicht gut)
◯	◯	◯	◯	◯	◯

Wurde Dein Herzfehler schon während der Schwangerschaft festgestellt (pränatal)?

Bitte Zutreffendes ankreuzen.

- ◯ Ja.
- ◯ Nein.
- ◯ Ich weiß es nicht.

Wie viel Zeit verbrachtest Du vor der Einschulung im Krankenhaus aufgrund des Herzfehlers?

Ein Beispiel: Anna verbringt in ihrem ersten Lebensjahr zwei Monate wegen ihres angeborenen Herzfehlers im Krankenhaus, als sie vier Jahre alt ist muss sie nochmal für einen Monat in der Klinik sein. Anna kreuzt also die zweite Antwort an, da sie insgesamt 3 Monate vor ihrer Einschulung im Krankenhaus verbracht hat.

Bitte Zutreffendes ankreuzen.

- ◯ Weniger als 1 Monat.
- ◯ 1 bis 3 Monate.
- ◯ 4 bis 6 Monate.
- ◯ 7 bis 9 Monate.
- ◯ 10 bis 12 Monate.
- ◯ 1 bis 2 Jahre.
- ◯ Mehr als 2 Jahre.
- ◯ Ich weiß es nicht.

Sind psychomotorische Erkrankungen oder psychomotorische Auffälligkeiten z. B. im Rahmen einer U-Untersuchung festgestellt worden?

Bitte Zutreffendes ankreuzen. Es sind Mehrfachantworten möglich.

- ☐ Ja, eine hyperkinetische Störung/eine Aufmerksamkeitsdefizitstörung wie z. B. ADHS.
- ☐ Ja, eine psychomotorischen Entwicklungsstörung.
- ☐ Ja, eine Intelligenzstörung.
- ☐ Ja, eine Störung des Sozialverhaltens.
- ☐ Ja, eine emotionale Störung.
- ☐ Ja, eine Depression.
- ☐ Ja, eine Angststörung.
- ☐ Ja, eine Lernstörung/Lernschwäche wie z. B. eine Leseschwäche oder Rechenschwäche.
- ☐ Ja, eine Sprech-/Sprachstörung.
- ☐ Ja, folgende Störung(en):
- ☐ Nein.
- ☐ Ich weiß es nicht.

Hast Du Epilepsie?
Bitte Zutreffendes ankreuzen.

- ◯ Ja.
- ◯ Nein.

Trägst Du eine Sehhilfe (Brille oder Kontaktlinsen)?
Bitte Zutreffendes ankreuzen.

- ◯ Ja.
- ◯ Nein.

Trägst Du ein Hörgerät?
Bitte Zutreffendes ankreuzen.

- ◯ Ja.
- ◯ Nein.

Brauchst Du eine Gehhilfe (z. B. Krücke, Rollator, Rollstuhl)?
Bitte Zutreffendes ankreuzen.

- ◯ Ja.
- ◯ Nein.

Wurde bei Dir schon einmal ein MRT von Deinem Kopf gemacht?
Bitte Zutreffendes ankreuzen.

- ◯ Ja.
- ◯ Nein.
- ◯ Ich weiß es nicht.

Wenn bei Dir schon einmal ein MRT vom Kopf gemacht wurde, weißt Du noch wie alt Du da warst und was der Grund dafür war?
Wenn Du weißt, warum das MRT gemacht wurde und wie alt Du bei der Untersuchung gewesen bist, dann wäre es super, wenn Du uns das hier kurz aufschreibst. Wenn noch nie ein MRT gemacht wurde, brauchst Du hier nichts eintragen.

Bitte beschreibe hier alle Medikamente, die Du derzeit einnimmst.

Bitte mache Angaben zu allen Medikamenten, die Du regelmäßig einnimmst. Wenn Du es weißt, gib bitte auch an, wann Du welches Medikament einnimmst (z. B. einmal, zweimal oder dreimal pro Tag) und wenn Du dann auch noch auswählst, wie viel von welchem Medikament Du einnimmst (z. B. eine Tablette/Kapsel, 10 Tropfen), wäre das toll.

Hinweis 1:
Wenn Du keine Medikamente einnimmst, kannst Du die nachfolgenden Fragen überspringen (scrolle dazu nach unten und klicke einfach auf „Weiter").
Hinweis 2:
Wenn Du z. B. nur ein Medikament regelmäßig einnimmst, fülle nur Medikament 1 aus, lass die nachfolgenden Fragen leer (scrolle dazu nach unten und klicke einfach auf „Weiter").

Medikament 1
Name des Medikaments:

Wann bzw. wie oft nimmst Du das Medikament ein?

- ◯ einmal am Tag (nur morgens, nur mittags oder nur abends).
- ◯ zweimal am Tag (morgens und mittags oder mittags und abends).
- ◯ dreimal am Tag (morgens, mittags und abends).
- ◯ häufiger als dreimal pro Tag.
- ◯ ich weiß es nicht.

Aus welchem Grund nimmst Du das Medikament ein?
Bitte schreibe hier kurz auf, warum Du dieses Medikament einnimmst. Wenn Du nicht weißt, warum oder wofür Du dieses Medikament einnimmst, schreibe einfach „Ich weiß es nicht" hin.

Wie nimmst Du das Medikament zu Dir?
Bitte gib an, in welcher Form Du das Medikament einnimmst.

- als Tablette/Kapsel.
- als Tropfen.
- als Spritze.
- in einer anderen Form, nämlich:

Wenn Du das Medikament in Tablettenform oder als Kapsel einnimmst, wie viele Tabletten/Kapseln nimmst Du dann insgesamt pro Tag ein?

- ¼ Tablette/Kapsel.
- ½ Tablette/Kapsel.
- ¾ Tablette/Kapsel.
- 1 Tablette/Kapsel.
- 1½ Tabletten/Kapseln.
- 2 Tabletten.
- 2½ Tabletten/Kapseln.
- 3 Tabletten/Kapseln.
- mehr als 3 Tabletten/Kapseln.
- eine andere Dosis pro Tag, nämlich:
- ich weiß nicht, wie viele Tabletten ich pro Tag einnehme.

Wenn Du das Medikament als Tropfen einnimmst, wie viele Tropfen nimmst Du insgesamt pro Tag ein?

- 1-10 Tropfen.
- 11-20 Tropfen.
- 21-30 Tropfen.
- 31-40 Tropfen.
- 41-50 Tropfen.
- 51-60 Tropfen.
- 61-70 Tropfen.
- 71-80 Tropfen.
- mehr als 80 Tropfen.
- ich weiß nicht, wie viele Tropfen ich pro Tag einnehme.

Wenn Du das Medikament in Form einer Spritze oder anders (z. B. als Spray) einnimmst, weißt Du dann, welche Dosis Du insgesamt pro Tag einnimmst?
Bitte beschreibe hier kurz, in welcher Form und in welcher Dosis Du das Medikament einnimmst. Wenn Du nicht weißt, wie und/oder in welcher Form Du dieses Medikament einnimmst, schreibe einfach „Ich weiß es nicht" hin.

Wo bist Du während der Schulzeit überwiegend aufgewachsen?
Bitte Zutreffendes ankreuzen.

- Bei meinen leiblichen Eltern.
- Bei meiner Mutter.
- Bei meinem Vater.
- Bei meinen Adoptiveltern.
- Bei Verwandten.
- In einer Pflegefamilie.
- In einer staatlichen Institution.
- Woanders, nämlich:

Hast Du regelmäßig eine Form von Frühförderung wie z. B. Logopädie, Physiotherapie oder Ergotherapie vor der Einschulung erhalten?
Bitte Zutreffendes ankreuzen.

- ○ Ja, für weniger als 3 Monate.
- ○ Ja, für 3 bis 6 Monate.
- ○ Ja, für 6 bis 12 Monate.
- ○ Ja, für 1 bis 2 Jahre.
- ○ Ja, für über 2 Jahre.
- ○ Ja, aber ich weiß nicht für wie lange.
- ○ Nein.
- ○ Ich weiß es nicht.

Wenn Du vor der Einschulung regelmäßig Frühförderung erhalten hast, kreuze diese bitte an.
Bitte Zutreffendes ankreuzen. Es sind Mehrfachantworten möglich.

- ☐ Logopädie
- ☐ Physiotherapie
- ☐ Psychotherapie
- ☐ Ergotherapie.
- ☐ Eine andere Frühförderung, nämlich: ________
- ☐ Keine regelmäßige Frühförderung vor der Einschulung.
- ☐ Ich weiß es nicht.

Mit wie vielen Jahren erfolgte die Einschulung?
Bitte Zutreffendes ankreuzen.

- ○ Meine Einschulung erfolgte im Altern von (Angabe in Jahren): ____
- ○ Ich weiß es nicht.

Auf welche der folgenden Schulformen erfolgte die Einschulung?
Bitte Zutreffendes ankreuzen.

- ○ Auf eine Grundschule.
- ○ Auf eine Grundschule in einer Integrationsklasse.
- ○ Auf eine Förderschule/Sonderschule.
- ○ Auf eine alternative Schulform wie z. B. eine Waldorfschule, Montessori- Schule oder Internat.
- ○ Ich weiß es nicht.

Mit wie vielen Jahren erfolgte der Wechsel auf eine weiterführende Schule?
Bitte Zutreffendes ankreuzen.

- ○ Mein Wechsel auf eine weiterführende Schule erfolgte im Altern von (Angabe in Jahren): ____
- ○ Es ist noch kein Wechsel auf eine weiterführende Schule erfolgt.
- ○ Ich weiß es nicht.

Welche weiterführende Schulform hast Du direkt nach der Grundschule besucht?
Bitte Zutreffendes ankreuzen.

- ○ Ich gehe noch zur Grundschule.
- ○ Ich habe nach der Grundschule keine weiterführende Schule besucht.
- ○ Hauptschule.
- ○ Realschule.
- ○ Schulform mit mehreren Bildungsgängen.
- ○ Integrierte Gesamtschule.
- ○ Gymnasium.
- ○ Andere, und zwar: ________
- ○ Ich weiß es nicht.

Bist oder warst Du vom Sportunterricht befreit?
Bitte Zutreffendes ankreuzen.

- ○ Ja, aufgrund einer ärztlichen Empfehlung.
- ○ Ja, die Teilnahme erfolgte nach meinem eigenem Ermessen.
- ○ Nein.

Musstest Du jemals eine Klasse wiederholen?
Bitte Zutreffendes ankreuzen.

- ○ Ja, ich musste eine Klasse wiederholen, nämlich folgende (bitte Klassenstufe eintragen, z. B. "8", wenn Du die achte Klasse wiederholt hast): ____
- ○ Ja, aber ich weiß nicht welche Klasse ich wiederholen musste.
- ○ Ja, ich musste mehrere Klassen wiederholen.
- ○ Nein, ich musste keine Klasse wiederholen.
- ○ Nein, ich musste keine Klasse wiederholen, sondern habe sogar eine Klasse übersprungen.

Wie viel Schulzeit hast Du aufgrund Deines Herzfehlers insgesamt verpasst?
Bitte Zutreffendes ankreuzen.

- ◯ Weniger als 1 Monat.
- ◯ 1 bis 3 Monate.
- ◯ 3 bis 6 Monate.
- ◯ 6 bis 9 Monate.
- ◯ 9 bis 12 Monate.
- ◯ 1 bis 2 Jahre.
- ◯ Mehr als 2 Jahre.
- ◯ Ich weiß es nicht.

Seitdem Du in die Schule gehst, hast Du schon einmal mindestens 3 Monate lang spezielle Fördermaßnahmen erhalten?
Bitte Zutreffendes ankreuzen. Es sind Mehrfachantworten möglich.

- ☐ Ja, Förderunterricht in der Schule.
- ☐ Ja, außerschulischen Nachhilfeunterricht.
- ☐ Ja, aber ich weiß nicht genau was für einen Förderunterricht.
- ☐ Nein, keine spezielle Fördermaßnahmen.
- ☐ Ich weiß es nicht.
- ☐ Ja, die folgenden Fördermaßnahmen: ______

Mit welchem Notendurchschnitt hast Du das letzte Schuljahr abgeschlossen?

- ◯ Mit folgendem Notendurchschnitt habe ich das letzte Schuljahr abgeschlossen: ______
- ◯ An meiner Schule gibt es (noch) keine Schulnoten auf dem Zeugnis
- ◯ Ich habe keine Noten bekommen, da ich (z. B. aus gesundheitlichen Gründen) zu viel Unterricht im letzten Schuljahr verpasst habe.
- ◯ Ich weiß es nicht.

In welche Klasse gehst Du im Moment?

- ◯ Im Moment besuche ich folgende Klasse: ______
- ◯ Ich weiß es nicht.

Welchen höchsten allgemeinbildenden Schulabschluss hat Deine Mutter?
Bitte Zutreffendes ankreuzen.

- ◯ Keinen Schulabschluss.
- ◯ Hauptschulabschluss/Volksschulabschluss.
- ◯ Realschulabschluss/Abschluss der polytechnischen Oberschule.
- ◯ Fachabitur/Fachgebundene Hochschulreife.
- ◯ Abitur/Allgemeine Hochschulreife.
- ◯ Einen anderen Schulabschluss, und zwar: ______
- ◯ Ich weiß es nicht.

Welchen höchsten beruflichen Ausbildungsabschluss hat Deine Mutter?
Bitte Zutreffendes ankreuzen.

- ◯ Keinen beruflichen Abschluss und ist nicht in einer beruflichen Ausbildung.
- ◯ Noch in einer beruflichen oder universitären Ausbildung (Berufsvorbereitungsjahr, Auszubildender, Praktikant, Student).
- ◯ Betriebliche Berufsausbildung (Lehre) abgeschlossen.
- ◯ Ausbildung an einer Fach-, Meister-, Technikerschule, Berufs-oder Fachakademie abgeschlossen.
- ◯ Bachelor an einer Fachhochschule oder an einer Universität.
- ◯ Master/Diplom an einer Fachhochschule.
- ◯ Master/Diplom/Magister/Staatsexamen/Promotion an einer Universität.
- ◯ Einen anderen beruflichen Abschluss, und zwar: ______
- ◯ Ich weiß es nicht.

Welche Erwerbssituation passt zu Deiner Mutter? Bitte beachte, dass unter Erwerbstätigkeit jede bezahlte bzw. mit einem Einkommen verbundene Tätigkeit verstanden wird.
Bitte Zutreffendes ankreuzen.

- ◯ Vollzeiterwerbstätig.
- ◯ Teilzeiterwerbstätig.
- ◯ Geringfügig erwerbstätig, 450-Euro-Job, Minijob.
- ◯ Nicht erwerbstätig.
- ◯ Ich weiß es nicht.

Wenn Deine Mutter nicht vollzeit- oder teilzeiterwerbstätig ist: Welche Bezeichnung passt zu Deiner Mutter am besten?
Bitte Zutreffendes ankreuzen.

- Meine Mutter ist voll- oder teilzeiterwerbstätig.
- Schülerin an einer allgemeinbildenden Schule.
- Studentin.
- Rentnerin oder Pensionärin.
- Arbeitsuchend.
- Dauerhaft erwerbsunfähig.
- Hausfrau.
- Sonstige und zwar:
- Ich weiß es nicht.

Welchen höchsten allgemeinbildenden Schulabschluss hat Dein Vater?
Bitte Zutreffendes ankreuzen.

- Keinen Schulabschluss.
- Hauptschulabschluss/Volksschulabschluss.
- Realschulabschluss/Abschluss der polytechnischen Oberschule.
- Fachabitur/Fachgebundene Hochschulreife.
- Abitur/Allgemeine Hochschulreife.
- Einen anderen Schulabschluss, und zwar:
- Ich weiß es nicht.

Welchen höchsten beruflichen Ausbildungsabschluss hat Dein Vater?
Bitte Zutreffendes ankreuzen.

- Keinen beruflichen Abschluss und ist nicht in einer beruflichen Ausbildung.
- Noch in einer beruflichen oder universitären Ausbildung (Berufsvorbereitungsjahr, Auszubildender, Praktikant, Student).
- Betriebliche Berufsausbildung (Lehre) abgeschlossen.
- Ausbildung an einer Fach-, Meister-, Technikerschule, Berufs-oder Fachakademie abgeschlossen.
- Bachelor an einer Fachhochschule oder an einer Universität.
- Master/Diplom an einer Fachhochschule.
- Master/Diplom/Magister/Staatsexamen/Promotion an einer Universität.
- Einen anderen beruflichen Abschluss, und zwar:
- Ich weiß es nicht.

Welche Erwerbssituation passt zu Deinem Vater? Bitte beachte, dass unter Erwerbstätigkeit jede bezahlte bzw. mit einem Einkommen verbundene Tätigkeit verstanden wird.
Bitte Zutreffendes ankreuzen.

- Vollzeiterwerbstätig.
- Teilzeiterwerbstätig.
- Geringfügig erwerbstätig, 450-Euro-Job, Minijob.
- Nicht erwerbstätig.

Wenn Dein Vater nicht vollzeit- oder teilzeiterwerbstätig ist: Welche Bezeichnung passt zu Deinem Vater am besten?
Bitte Zutreffendes ankreuzen.

- Mein Vater ist voll- oder teilzeiterwerbstätig.
- Schüler an einer allgemeinbildenden Schule.
- Student.
- Rentner oder Pensionär.
- Arbeitsuchend.
- Dauerhaft erwerbsunfähig.
- Hausmann.
- Sonstige, und zwar:
- Ich weiß es nicht.
