# Supplementary material for: Microcephaly is associated with impaired educational development in children with congenital heart disease
Source: Front Cardiovasc Med. 2022 Oct 6;9:917507. doi: 10.3389/fcvm.2022.917507 (PMC9584804; doi:10.3389/fcvm.2022.917507)
Supplement: Supplementary file 3 [file Data_Sheet_2.docx]

**APPENDIX**

**List of the German Competence Network for Congenital Heart Defects Investigators**

Gunter Kerst, Majed Kanaan, Klinik für Kinderkardiologie; Corinna Lebherz, Klinik für Kardiologie, André Rüffer, Herzchirurgie für Kinder und Erwachsene mit angeborenen Herzfehlern, Universitätsklinikum Aachen; Aachen.

Dimitrios Gkalpakiotis, Praxis für Kinderkardiologie; Aachen.

Andrea Schedifka, Praxis für Kinder‐ und Jugendmedizin, Kinderkardiologie; Ahrensfelde.

Gernot Buheitel, Joachim Streble, II. Klinik für Kinder und Jugendliche, Universitätsklinikum Augsburg; Augsburg.

Rainer Willing, Klinik für Kinder‐ und Jugendmedizin, Kinderkardiologie, Ubbo‐Emmius‐Klinik; Aurich.

Stephan Schubert, Kai Thorsten Laser, Karl‐Otto Dubowy Kinderherzzentrum/Zentrum für angeborene Herzfehler, Klinik für Kinderkardiologie und angeborene Herzfehler; Eugen Sandica, Kinderherzzentrum/Zentrum für angeborene Herzfehler, Klinik für Kinderherzchirurgie und angeborene Herzfehler, Herz‐ und Diabeteszentrum NRW; Bad Oeynhausen.

Burkhard Trusen, Praxis Kinder‐ und Jugendmedizin, Kinderkardiologe; Bamberg.

Felix Berger, Oliver Miera, Stanislav Ovroutski, Katharina Schmitt, Klinik für angeborene Herzfehler und Kinderkardiologie; Joachim Photiadis, Klinik für die Chirurgie Angeborener Herzfehler/Kinderherzchirurgie, Deutsches Herzzentrum Berlin; Berlin.

Felix Berger, Bernd Opgen‐Rhein, Katja Weiss, Sabine Klaassen, Klinik für Pädiatrie mit Schwerpunkt Kardiologie, Charité ‐ Universitätsmedizin Berlin, Campus Virchow‐Klinikum; Berlin.

Christoph Berns, Praxis für Kinderheilkunde, Jugendmedizin und Kinderkardiologie; Berlin.

Thomas Boeckel, Guido Haverkämper, Praxis für Kinder‐ und Jugendmedizin, Kinderkardiologie; Berlin.

Andreas Kästner, Heike Koch, Björn Peters, Gemeinschaftspraxis für Pädiatrische Kardiologie; Berlin.

Florian Schmidt, Praxis für Kinder‐ und Jugendmedizin, Kinderkardiologie; Berlin.

Jens Timme, Konstanze Engel, Birgit Franzbach, Gabriela Senft, Facharztpraxis für Kinderkardiologie und Erwachsene mit angeborenem Herzfehler; Berlin.

Frank Beyer, Praxis für Kinder‐ und Jugendmedizin, Kinderkardiologie; Bielefeld.

Klaus Winter, Klinik für Kinder‐ und Jugendmedizin, St.‐Agnes‐Hospital; Bocholt.

Johannes Breuer, Martin Schneider, Zentrum für Kinderheilkunde, Abteilung für Kinderkardiologie; Boulos Asfour, Klinik und Poliklinik für Herzchirurgie, Universitätsklinikum Bonn; Bonn.

Jens Bahlmann, Eberhard Griese, Kinderkardiologische Gemeinschaftspraxis; Braunschweig.

Trong Phi Lê, Klinik für strukturelle und angeborene Herzfehler/ Kinderkardiologie, Klinikum Links der Weser; Bremen.

Joachim Hebe, Jan‐Hendrik Nürnberg, Elektrophysiologie Bremen, Zentrum Bremen am Klinikum Links der Weser; Bremen.

Annette Magsaam, Praxis für Kinderkardiologie und Angeborene Herzfehler; Bremen.

Ronald Müller, Praxis für Angeborene Herzfehler/ Kinderkardiologie; Bremen.

Ludger Potthoff, Praxis Celler Centrum für Kinder‐ und Jugendmedizin, Kinderkardiologie; Celle.

Renate Voigt, Praxis für Kinder‐ und Jugendmedizin, Kinderkardiologie; Chemnitz.

Tim Krüger, Kinderarzt‐Praxis Ilmenau/Coburg, Praxis für Kinder‐ und Jugendmedizin, Kinderkardiologie; Coburg.

Hubert Gerleve, Ulrich Kleideiter, Kinder‐ und Jugendklinik, Christophorus‐Kliniken Coesfeld; Coesfeld.

Dirk Schneider‐Kulla, Klinik für Pädiatrie/Kinder‐ und Jugendheilkunde, Kinderkardiologie; Jürgen Krülls‐Münch, I. Medizinische Klinik, Klinik für Kardiologie, Angiologie und internistische Intensivtherapie, Carl‐Thiem‐Klinikum Cottbus; Cottbus.

Thomas Menke, Kinderkardiologie, Vestische Kinder‐ und Jugendklinik Datteln; Datteln.

Martin Lehn, Praxis für Kinder‐ und Jugendkardiologie und für Erwachsene mit angeborenen Herzfehlbildungen; Dortmund.

Antje Heilmann, Helge Tomczak, Praxis für Kinderkardiologie, Kinderzentrum Dresden‐Friedrichstadt; Dresden.

Gleb Tarusinov, Klinik für Kinderkardiologie ‐ Angeborene Herzfehler; Michael Scheid, Kinderherzchirurgie und Chirurgie für angeborene Herzfehler, Herzzentrum Duisburg; Duisburg.

Ertan Mayatepek, Frank Pillekamp, Klinik für Allgemeine Pädiatrie, Neonatologie und Kinderkardiologie; Artur Lichtenberg, Klinik für Kardiovaskuläre Chirurgie, Universitätsklinikum Düsseldorf; Düsseldorf.

Christiane Terpeluk, Praxis für Kinder‐ und Jugendmedizin, Kinderkardiologie; Ehingen.

Bruno Kolterer, Kinderkardiologische Schwerpunktpraxis; Erfurt.

Sven Dittrich, Kinderkardiologische Abteilung; Ulrike Gundlach, Medizinische Klinik 2 ‐Kardiologie und Angiologie; Robert Cesnjevar, Kinderherzchirurgische Abteilung, Universitätsklinikum Erlangen, Friedrich‐Alexander‐Universität Erlangen‐Nürnberg; Erlangen.

Carsten Müntjes, Klinik für Kinderheilkunde III, Abteilung für Pädiatrische Kardiologie Universitätsklinikum Essen; Essen.

Geert Morf, Praxis für Kinder‐ und Jugendmedizin und EMAH, Kinderkardiologie; Flensburg.

Anoosh Esmaeili, Klinik für Kinder‐ und Jugendmedizin, Kinderkardiologie, Universitätsklinikum Frankfurt; Frankfurt.

Stephan Backhoff, Praxis für Kinderkardiologie/angeborene Herzerkrankungen; Frankfurt.

Brigitte Stiller, Zentrum für Kinder‐ und Jugendmedizin, Klinik für Angeborene Herzfehler/Pädiatrische Kardiologie; Friedhelm Beyersdorf, Klinik für Herz‐ und Gefäßchirurgie; Johannes Kroll, Klinik für Herz‐ und Gefäßchirurgie, Sektion Kinderherzchirurgie, Universitäts‐Herzzentrum Freiburg Bad Krozingen; Freiburg.

Nicole Häffner, Praxis für Kinder‐ und Jugendmedizin, Kinderkardiologie; Freiburg.

Jannos Siaplaouras, Praxis für Kinder‐ und Jugendmedizin, Kinderkardiologie, Erwachsene mit angeborenem Herzfehler, Bluttransfusionswesen am Herz‐Jesu‐Krankenhaus; Fulda.

Antje Masri‐Zada, Praxis für Kardiologie; Gera.

Christian Jux, Klinik für Kinderkardiologie und angeborene Herzfehler; Andreas Böning, Hakan Akintürk, Klinik für Herz‐, Kinderherz‐ und Gefäßchirurgie, Universitätsklinikum Gießen und Marburg; Gießen.

Thomas Paul, Matthias Sigler, Klinik für Pädiatrische Kardiologie und Intensivmedizin mit Neonatologie und Pädiatrischer Pneumologie; Theodor Tirilomis, Klinik für Thorax‐, Herzund Gefäßchirurgie – Schwerpunkt Kinderherzchirurgie, Universitätsklinikum Göttingen; Göttingen.

Gabriele Schürer, Praxis für Kinder‐ und Jugendmedizin, Kinderkardiologie; Greiz.

Johannes Hartmann, Schwerpunktpraxis für Kinder‐ und Jugendkardiologie; Hagen.

Ralph Grabitz, Uta Liebaug, Universitätsklinik und Poliklinik für Pädiatrische Kardiologie, Universitätsklinikum Halle (Saale); Halle.

Claudius Rotzsch, Kinderkardiologische Praxis; Halle.

Rainer Kozlik‐Feldmann, Carsten Rickers, Thomas Mir, Michael Hübler, Jörg Sachweh, Kinderkardiologie/Herzchirurgie für angeborene Herzfehler, Universitäres Herz‐ und Gefäßzentrum UKE Hamburg; Hamburg.

Stefan Renz, Andreas Schemm, Praxis für Kinder‐ und Jugendmedizin, Kinderkardiologie und EMAH; Hamburg.

Bernd Friedrich, Otmar Schlobohm, Kinder‐ und Jugendarztpraxis, Kinderkardiologie; Hamburg.

Dietmar Böthig, Burkhard Wermter, Andrea Kelter‐Klöpping, Klinik für Pädiatrische Kardiologie und Intensivmedizin; Alexander Horke, Chirurgie angeborener Herzfehler; Johann Bauersachs, Mechthild Westhoff‐Bleck, Klinik für Kardiologie und Angiologie, Medizinische Hochschule Hannover; Hannover.

Matthias Gorenflo, Zentrum für Kinder‐ und Jugendmedizin Pädiatrische Kardiologie/Angeborene Herzfehler, Matthias Karck, Tsvetomir Loukanov, Klinik für Herzchirurgie, Universitätsklinikum Heidelberg; Heidelberg.

Hermann Schrüfer, Praxis für Kinder‐ und Jugendmedizin, Kinderkardiologie; Hettstadt.

Martin Wilken, Kinderarzt‐Praxis Hof/Nail; Praxis für Kinder‐ und Jugendmedizin, Kinderkardiologie; Hof.

Hashim Abdul‐Khaliq, Tanja Rädle‐Hurst, Axel Rentzsch, Klinik für Pädiatrische Kardiologie; Hans‐Joachim Schäfers, Klinik für Thorax‐ und Herz‐Gefäß‐Chirurgie, Universitätsklinikum des Saarlandes; Homburg.

Hagen Reichert, Praxis für Kinder‐ und Jugendmedizin, Kinderkardiologie; Reisemedizin, Gelbfieberimpfstelle; Homburg.

Daniel Vilser, Klinik für Kinder‐ und Jugendmedizin, Sektion Kardiologie, Universitätsklinikum Jena; Jena.

Thomas Kriebel, Klinik für Kinder‐ und Jugendmedizin, Kinderkardiologie, Westpfalz‐Klinikum; Kaiserslautern.

Arnulf Boysen, Schwerpunktpraxis für angeborene Herzfehler; Karlsruhe.

Anselm Uebing, Inga Voges, Klinik für angeborene Herzfehler und Kinderkardiologie; Tim Attmann, Joachim Thomas Cremer, Jens Scheewe, Klinik für Herz‐ und Gefäßchirurgie, Universitätsklinikum Schleswig‐Holstein; Kiel.

Regina Buchholz‐Berdau, Peter Möller, Gemeinschaftspraxis der Kinder‐ und Jugendärzte und Kinderkardiologen; Kiel.

Thorsten Horter, Schwerpunktpraxis für Kinder‐ und Jugendkardiologie; Kiel.

Konrad Brockmeier, Klinik und Poliklinik für Kinderkardiologie, Gerardus B. W. EBennink, Klinik für Herz‐ und Thoraxchirurgie, Schwerpunkt Kinderherzchirurgie, Stephan Baldus, Klinik III für Innere Medizin, Herzzentrum Universitätsklinikum Köln; Köln.

Alex Gillor, Praxis für Kinder‐ und Jugendkardiologie; Köln.

Tim Niehues, Wolfgang Lawrenz, Zentrum für Kinder‐ und Jugendmedizin, Kinderkardiologie, HELIOS Klinikum Krefeld; Krefeld.

Steffen Leidig, Praxis für Kinder‐ und Jugendmedizin, Kinderkardiologie; Lauf.

Ingo Dähnert, Frank‐Thomas Riede, Universitätsklinik für Kinderkardiologie; Martin Kostelka, Universitätsklinik für Herzchirurgie, Kinderherzchirurgie, Herzzentrum Leipzig; Leipzig.

Liane Kändler, Medizinisches Versorgungszentrum Jessen, Außenstelle Wittenberg und Klinik für Kinder‐ und Jugendmedizin, Paul Gerhardt Diakonie und Pflege GmbH; Lutherstadt Wittenberg.

Martin Bethge, Stefan Köster, Praxis für Kinder‐ und Jugendmedizin, Kinderkardiologie und EMAH; Lübeck.

Christoph Schröder, Praxis für Kinderkardiologie, Kinderpneumologie, Erwachsene mit angeborenen Herzfehlern; Lüneburg.

Jens Karstedt, Kardiologische Schwerpunkpraxis für Kinder und Jugendliche am Klinikum Magdeburg; Magdeburg.

Uwe Seitz, Praxis für Kinder‐ Jugendmedizin, Kinderkardiologie; Maintal.

Christoph Kampmann, Zentrum für Kinder‐ und Jugendmedizin, Abteilung für Kinderkardiologie, Daniel‐Sebastian Dohle, Klinik und Poliklinik für Herz‐, Thorax‐ und Gefäßchirurgie, Universitätsmedizin der Johannes Gutenberg‐Universität Mainz; Mainz.

Frank Stahl, Praxis für Kinder‐ und Jugendkardiologie, arterielle Hypertonie bei Kindern und Jugendlichen, Erwachsene mit angeborenen Herzfehlern; Mannheim.

Mojtaba Abedini, Praxis für Kinder‐ und Jugendkardiologie am Universitätsklinikum Gießen und Marburg; Marburg.

Joachim Müller‐Scholden, Praxis für Kinder‐ und Jugendmedizin, Kinderkardiologie; Marktheidenfeld.

Peter Ewert, Alfred Hager, Michael Huntgeburth, Harald Kaemmerer, Nicole Nagdyman, Jörg Schoetzau, Oktay Tutarel, Klinik für Kinderkardiologie und Angeborene Herzfehler; Rüdiger Lange, Klinik für Herz‐ und Gefäßchirurgie; Jürgen Hörer, Klinik für Chirurgie angeborener Herzfehler und Kinderherzchirurgie Deutsches Herzzentrum München; München.

Nikolaus AHaas, Abteilung Kinderkardiologie und Pädiatrische Intensivmedizin; Jürgen Hörer, Herzchirurgische Klinik und Poliklinik, Sektion Kinderherzchirurgie; Klinikum der Ludwig‐Maximilians‐Universität, Campus Großhadern; München.

Michael Hauser, Praxis für Kinder‐ und Jugendkardiologie und Erwachsene mit angeborenen Herzfehlern; München.

Alexander Roithmaier, Praxis für Kinder‐ und Jugendmedizin, Schwerpunktpraxis für Kinderund Jugendkardiologie; München.

Hans‐Gerd Kehl, Astrid Lammers, Klinik für Kinder‐ und Jugendmedizin – Pädiatrische Kardiologie, Edward Malec, Department für Herz‐ und Thoraxchirurgie, Abteilung Kinderherzchirurgie; Helmut Baumgartner, Gerhard Diller, Klinik und Poliklinik für Erwachsene mit angeborenen (EMAH) und erworbenen Herzfehlern, Universitätsklinikum Münster; Münster.

Roswitha Bahle, Praxis für Kinder‐ und Jugendmedizin, Kinderkardiologie; Neubrandenburg.

Gerald Hofner, Praxis für Kinder‐ und Jugendmedizin, Kinderkardiologie; Neudrossenfeld.

Stefan Zink, Praxis für Kinder‐ und Jugendmedizin, Kinderkardiologie; Nürnberg.

Roland Reif, Helmut Singer; Gemeinschaftspraxis für Kinder‐ und Jugendmedizin, Kinderkardiologie, Allergologie, Asthmatraining, Psychotherapie; Nürnberg.

Christoph Parlasca, Klinik für Kinder‐ und Jugendmedizin, Evangelisches Krankenhaus Oberhausen; Oberhausen.

Matthias WFreund, Michael Schumacher, Universitätsklinik für Kinder‐ und Jugendmedizin, Klinik für Neonatologie, Intensivmedizin und Kinderkardiologie, Klinikum Oldenburg ‐Elisabeth‐Kinderkrankenhaus; Oldenburg.

Oliver Dewald, Universitätsklinik für Herzchirurgie, Klinikum Oldenburg; Oldenburg.

Christine Darrelmann, Gemeinschaftspraxis für Kinder‐ und Jugendmedizin, Kinderkardiologie; Oldenburg.

Olaf Willmann, Praxis für Kinder‐ und Jugendmedizin, Kinderkardiologie; Osnabrück.

Norbert Schmiedl, Praxis für Kinderkardiologie; Passau.

Peter Quick, Praxis für Kinder‐ und Jugendmedizin, Kinderkardiologie; Plauen.

Dirk Hillebrand, Praxis für Kinder‐ und Jugendmedizin, Schwerpunktpraxis Kinderkardiologie, Angeborene Herzfehler; Pinneberg.

Stephan Michele Eiselt, Praxis für Kinder‐ und Jugendmedizin, Kinderkardiologie und EMAH; Reinbek.

Torsten Nekarda, Klinik für Kinder‐ und Jugendmedizin, Agaplesion Diakonieklinikum Rotenburg; Rotenburg.

Michael Eberhard, Praxis für Kinder‐ und Jugendmedizin, Kinderkardiologie; Rottweil.

Georg Baier, Praxis für Kinder‐ und Jugendmedizin, Kinderkardiologie; Schwabach.

Frank Uhlemann, Zentrum für angeborene Herzfehler, Olgahospital; Stuttgart.

Ioannis Tzanavaros, Chirurgie für angeborene Herzfehler/Kinderherzchirurgie, Sana Herzchirurgie Stuttgart; Stuttgart.

Alexander Beyer, Gudrun Binz, Steffen Hess, Thomas Teufel, Kinderkardiologische Praxis Stuttgart/EMAH‐Schwerpunktpraxis; Stuttgart.

Ronald‐Peter Handke, Praxis für Kinder‐ und Jugendmedizin, Kinderkardiologie; Trier.

Michael Hofbeck, Renate Kaulitz, Ludger Sieverding, Kinderheilkunde II ‐ Kinderkardiologie, Intensivmedizin und Pulmologie; Christian Schlensak, Thorax‐, Herz‐ und Gefäßchirurgie, Migdat Mustafi, Sektion Chirurgie angeborener Herzfehler – Kinderherzchirurgie, Universitätsklinikum Tübingen; Tübingen.

Christian Apitz, Michael Kaestner, Klinik für Kinder‐ und Jugendmedizin, Sektion Pädiatrische Kardiologie, Universitätsklinikum Ulm; Ulm.

Jürgen Holtvogt, Klinik für Kinder‐ und Jugendmedizin, Kinderkardiologie, St. Marienhospital Vechta; Vechta.

Carl‐Friedrich Wippermann, Praxis für Kinder‐ und Jugendmedizin, Kinderkardiologie; Walluf.

Sönke Hinz, Praxis für Kinder‐ und Jugendmedizin, Kinderkardiologie; Weyhe.

Andreas Heusch, Zentrum für Kinder‐ und Jugendmedizin, Abteilung Kinderkardiologie und ‐pneumologie, HELIOS Klinikum Wuppertal; Wuppertal.

Johannes Wirbelauer, Kinderklinik, Kinderkardiologie/ EMAH, Universitätsklinikum Würzburg; Würzburg.

Wolfgang Brosi, Praxis für Kinder‐ und Jugendmedizin, Kinderkardiologie und –pneumologie, Allergologie, Umweltmedizin, Asthma‐, Neurodermitis‐ und Anaphylaxietrainer; Würzburg.
